# Agaritine derived from *Agaricus blazei* Murrill induces apoptosis via mitochondrial membrane depolarization in hematological tumor cell lines

**DOI:** 10.20407/fmj.2022-021

**Published:** 2022-10-28

**Authors:** Atsushi Ogasawara, Hiroki Doi, Taei Matsui, Etsuko Tokunaga, Masao Amakawa, Hidehiko Akiyama

**Affiliations:** 1 Field of Clinical Laboratory Sciences, Fujita Health University, Graduate School of Health Sciences, Toyoake, Aichi, Japan; 2 Department of Medical Technology, Gifu University of Medical Science, Seki, Gifu, Japan

**Keywords:** *Agaricus blazei* Murrill, Agaritine (AGT), Apoptosis, Mitochondrial membrane depolarization

## Abstract

**Objectives:**

Agaritine (AGT) is a hydrazine-containing compound derived from the mushroom *Agaricus blazei* Murill. We previously reported the antitumor effect of AGT on hematological tumor cell lines and suggested that AGT induces apoptosis in U937 cells via caspase activation. However, the antitumor mechanism of AGT has not been fully understood.

**Methods:**

Four hematological tumor cell lines (K562, HL60, THP-1, H929) were used in this study. The cells were incubated in the presence of 50 μM AGT for 24 h and analyzed for cell viability, annexin V positivity, caspase-3/7 activity, mitochondrial membrane depolarization, cell cycle, DNA fragmentation, and the expression of mitochondrial membrane-associated proteins (Bax and cytochrome c).

**Results:**

In HL60, K562, and H929 cells, AGT reduced cell viability and increased annexin V- and dead cell-positive rates; however, it did not affect THP-1 cells. In K562 and HL60 cells, caspase-3/7 activity, mitochondrial membrane depolarization, and expression of mitochondrial membrane proteins, Bax and cytochrome c, were all increased by AGT. Cell cycle analysis showed that only K562 exhibited an increase in the proportion of cells in G_2_/M phase after the addition of AGT. DNA fragmentation was also observed after the addition of AGT.

**Conclusions:**

These results indicate that AGT induces apoptosis in K562 and HL60 cells, like U937 reported previously, but showed no effect on THP-1 cells. It was suggested that AGT-induced apoptosis involves the expression of Bax and cytochrome c via mitochondrial membrane depolarization.

## Introduction

Medicinal mushrooms are widely used in East Asia to treat a variety of diseases.^1^ Mushrooms have been shown to have many medicinal properties, such as anti-inflammatory, immunomodulatory, and antitumor effects.^2^

*Agaricus blazei* Murill (ABM) belongs to the Basidiomycetes family that grows naturally in the south of Sao Paulo State, Brazil.^3^ It is cultivated in Japan under the name *kawarihiratake* and has been reported to have many bioactive effects.^4^ Mushrooms of the genus *Agaricus* contain various minerals, vitamins, and dietary fibers as well as bioactive substances such as immune enhancers, which are believed to inhibit cancer progression and metastasis and are widely used in complementary cancer care.^5^ In particular, β-glucans (β-1,3 and β-1,6 bound glucans) from these mushrooms have been reported to act as immunostimulants against cancer cells.^6^

In a previous study, we found that the heat-stable and diffusible fractions of ABM exert antitumor activity in vitro. They directly reduced the viability of leukemia cell lines U937, MOLT4, HL60, and K562. Structural analysis revealed that the antitumor substance is agaritine (AGT), a hydrazine-containing compound.^7^ AGT has been reported to be present in 53 different species of the genus *Agaricus*.^8^ AGT is a genotoxic phenylhydrazine^9^ and is well known to be carcinogenic or tumorigenic,^10^ although its safety has remained unclear. However, we reported that AGT has neither a carcinogenic effect according to the *umu* test^7^ nor a toxic effect on normal lymphocytes.

Regarding the antitumor effect of AGT, we previously suggested that AGT induces apoptosis via caspase activation through cytochrome c release from mitochondria in the monocytic leukemia cell line U937.^11^ In this study, we further analyzed the antitumor effect of AGT using several hematological tumor cell lines and found that AGT induces apoptosis in K562 and HL60 cells through the expression of Bax and cytochrome c by mitochondrial membrane depolarization but shows no effect on THP-1 cells.

## Materials and Methods

### Materials

Pure-grade agaritine (AGT) monohydrate (C_12_H_17_N_3_O_4_·H_2_O) was obtained from FujiFilm-Wako (Osaka, Japan). AGT was dissolved in distilled water, filtered to 10 μg/mL, and used at a final concentration of 10–100 μM in the culture medium.

Cytosine arabinoside (Ara-C) and vincristine (VCR) were purchased from Sigma-Aldrich (St. Louis, MO, USA), dissolved in phosphate-buffered saline (PBS; 150 mM NaCl, 10 mM phosphate buffer, pH 7.2), and used at final concentrations of 40 and 0.1 μM, respectively, in the culture medium given that approximately 50% of THP-1 and H929 cells undergo apoptosis at these concentrations.^12^

### Cell culture

K562 cells (human chronic myelogenous leukemia cell line; EC89121407), HL60 cells (human promyelocytic leukemia cell line; EC98070106), THP-1 cells (human monocytic leukemia cell line; EC88081201), and NCI-H929 cells (human IgA-producing multiple myeloma cell line; EC95050415) were obtained from DS Pharma Biomedical (Osaka, Japan). RPMI-1640 medium (Sigma-Aldrich) supplemented with 10% fetal bovine serum (Nichirei Biosciences Inc., Tokyo, Japan), 100 U/mL penicillin, and 100 μg/mL streptomycin (Gibco, Carlsbad, CA, USA) was used as the culture medium. The cells were cultured at 37°C with 5% CO_2_.

### MTT assay

Cell viability was measured with a 3-(4,5-dimethylthiazol-2-yl)-2,5-diphenyltetrazolium bromide (MTT) assay kit (Cayman Chemical Company, Ann Arbor, USA). Each cell type was seeded at a concentration of 2×10^5^ cells/mL in a 24-well falcon dish (Becton Dickinson, Franklin Lakes, USA), to which each reagent was added. Then, 100 μL of cell suspension was seeded into 96-well plates (Becton Dickinson) and incubated at 37°C for 24 h. Then, 10 μL of MTT reagent was added to each well. After mixing gently, the cells were incubated at 37°C for 4 h in a CO_2_ incubator. Then, 100 μL of lysis buffer was added and mixed with the cell solution, followed by further incubation at 37°C for 12 h in a CO_2_ incubator. Finally, the optical density at 550 nm was measured using a microplate reader (Benchmark; Bio-Rad, Hercules, USA).

### Annexin V- and dead cell-positive rates

Rates of positivity for annexin V and dead cells were detected using the Muse^TM^ Annexin V and Dead Cell Assay Kit (Merck Millipore Corporation, Darmstadt, Germany). Annexin V can bind to phosphatidylserine (PS), a membrane component in early apoptosis, and 7-aminoactinomycin D (7-AAD) is a dead cell marker in late apoptosis to distinguish it from early apoptosis. These cells were seeded in a 24-well falcon dish (Becton Dickinson) at a concentration of 2×10^5^ cells/mL and incubated for 24 h after the addition of each reagent. Reagent-treated cells were collected by centrifugation (300×g at 4°C for 5 min) to remove the supernatant, resuspended in 100 μL of RPMI-1640 medium, and incubated with 100 μL of kit reagent for 20 min at room temperature. The rates of positivity for annexin V and dead cells were measured using a Muse Cell Analyzer (Merck Millipore Corporation).

### Caspase-3/7 activity

Caspase-3/7 activity was detected using the Muse^TM^ Caspase-3/7 Assay Kit (Merck Millipore Corporation). The cells were seeded in a 24-well falcon dish at a concentration of 2×10^5^ cells/mL and incubated for 24 h after the addition of each reagent. Reagent-treated cells were collected by centrifugation (300×g at 4°C for 5 min) to remove the supernatant, and suspended in 50 μL of RPMI-1640 medium. Then, these cells were incubated with 5 μL of caspase-3/7 reagent working solution (1 μL of Muse^TM^ Caspase3/7 Reagent and 7 μL of 1×PBS) for 30 min at room temperature in the dark. Finally, 150 μL of caspase 7-amino actinomycin D (7-AAD) working solution (2 μL of Muse^TM^ Caspase 7-AAD and 148 μL of 1×Assay Buffer BA) was added and caspase-3/7 expression was measured using a Muse Cell Analyzer.

### Mitochondrial membrane depolarization

Mitochondrial membrane depolarization was determined using the Muse^TM^ MitoPotential Kit (Merck Millipore Corporation). The cells were seeded in a 24-well falcon dish at a concentration of 2×10^5^ cells/mL and incubated for 24 h after the addition of each reagent. Reagent-treated cells were collected by centrifugation (300×g at 20°C for 5 min) to remove the supernatant, and then mixed with 100 μL of Assay Buffer and 95 μL of MitoPotential working solution (Muse^TM^ MitoPotential Dye diluted to 1:1000 in assay buffer). After incubating at 37°C for 20 min, Muse MitoPotential 7-AAD reagent (5 μL) was added to each tube, followed by vortexing for 3 to 5 s. After incubation at room temperature for 5 min, mitochondrial membrane depolarization was measured using the Muse Cell Analyzer.

### Cell cycle analysis

The cells were seeded in a 24-well falcon dish at a concentration of 2×10^5^ cells/mL and incubated for 24 h after the addition of each reagent. Reagent-treated cells were collected by centrifugation (300×g at 20°C for 5 min) to remove the supernatant, and resuspended in 50 μL of PBS and fixed with 450 μL of 80% ethanol for more than 3 h at –20°C. Cell pellets obtained by centrifugation (300×g, 5 min) were washed in 500 μL of PBS, incubated with 200 μL of Muse Cell Cycle Reagent in the dark for 30 min, and the cell cycle was measured using a Muse Cell Analyzer.

### DNA fragmentation analysis

The cells were seeded in a 24-well falcon dish at a concentration of 2×10^5^ cells/mL and incubated for 24 h after the addition of each reagent. Reagent-treated cells were centrifuged at 300×g for 5 min and washed once with PBS. The cell pellet was suspended in 100 μL of cell lysis buffer (10 mM Tris–HCl buffer, pH 7.4, containing 10 mM EDTA and 0.5% Triton X-100) and kept at 4°C for 10 min. The cell lysate was centrifuged at 16,000×g for 20 min. The supernatant (100 μL) was incubated with 2 μL of RNase A (20 mg/mL; Macherey-Nagel, Düren, Germany) at 37°C for 60 min, and then with 2 μL of proteinase K solution (20 mg/mL; Wako, Japan) at 37°C for 60 min. After adding 20 μL of 5 M NaCl and 120 μL of isopropyl alcohol, the mixture was kept at –30°C overnight. The precipitate was then collected by centrifugation at 16,000×g for 15 min and washed twice with 70% ethanol. After the removal of ethanol, samples were allowed to stand for 5 min on a clean bench to volatilize the remaining ethanol. Fragmented DNA was then dissolved in TE buffer (10 mM Tris–HCl, pH 7.4, and 1 mM EDTA) and subjected to 2% agarose gel electrophoresis at 100 V for 45 min. Fragmented DNA was stained with 0.5 μg/mL ethidium bromide solution (Genesee Scientific, San Diego, USA). The electrophoresed gel was photographed using Printgraph CMOS I (ATTO Co., Tokyo, Japan).

### Western blotting

The cells were seeded in a six-well falcon dish at a concentration of 1×10^6^ cells/3 mL and incubated for 24 h after the addition of each reagent. Reagent-treated cells were washed twice with 1×PBS at 200 g for 5 min at 4°C and the supernatant was then removed.

The cell lysates were obtained by incubating the cells for 15 min on ice with EzRIPA lysis buffer (ATTO Co., Tokyo, Japan) and centrifuging the lysates at 14,000×g for 10 min at 4°C. The protein concentration of the lysates was determined with the Takara BCA Protein Assay Kit (Takara Bio Inc., Koka, Japan). After mixing with the loading buffer and boiling at 95°C for 5 min, equal amounts of proteins were subjected to 15% sodium dodecyl sulfate polyacrylamide gel electrophoresis (SDS‐PAGE). Polyvinylidene difluoride (PVDF) membranes were used for protein transfer. The blocking of nonspecific binding sites on the membrane was achieved using 3% BSA dissolved in Tris‐buffered saline‐Tween (TBST) for 1 h at room temperature and then incubated with corresponding primary antibodies for 1.5 h at room temperature. The primary antibodies included anti-Bax (1:3,000, GTX109683; Funakoshi Co., Tokyo, Japan), anti-cytochrome c (1:1,000, GTX108585; Funakoshi Co.), and anti-beta tubulin (1:1,000, 10068-1-AP; Proteintech Japan, Co., Tokyo, Japan). After washing with TBST three times (10 min each), horseradish peroxidase-conjugated secondary antibody (1:3,000, 111-035-003; Jackson ImmunoResearch Laboratories, Inc., PA, USA) was added. The membranes were incubated at room temperature for 1.5 h and then washed with TBST three times (10 min each). A luminescent substrate (WSE-7110; ATTO Co., Tokyo, Japan) was used for generating the luminescence of the HRP-labeled antibody. The optical density of the protein was detected using a LuminoGraph I Imaging system (ATTO Co., Tokyo, Japan), and the intensity of bands was quantified by ImageJ software.

### Statistical analysis

Data were analyzed using Excel software and Student’s *t*-test was used to assess the statistical significance of differences between treatments. Results are expressed as mean±SD of three independent experiments. P<0.05 was considered statistically significant.

## Results

### MTT assay

**Figure 1:**
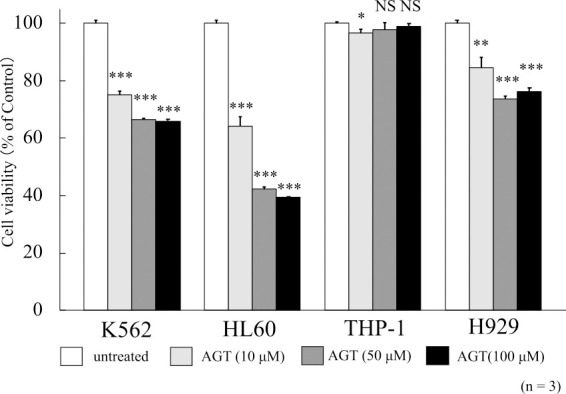
The cell viability after treatment for 24 h with AGT in four hematological tumor cell lines. The y-axis values of the cell viability histograms represent the optical density (550 nm) compared with the control (set as 100%), as measured by MTT assay. The optical density decreased after treatment with AGT compared with that of untreated cells in K562, HL60, and H929 cells, but its decrease was slight in THP-1 cells. The results are expressed as mean±SD of three independent experiments (NS, *P<0.05, **P<0.01, ***P<0.001, compared with untreated cells).

To evaluate the cell viability upon the addition of AGT (10, 50, or 100 μM), MTT assay was performed. The viability of K562, HL60, and H929 cells was reduced to 66.0%, 39.4%, and 76.3%, respectively, after 24 h of treatment with AGT (100 μM) compared with that of untreated cells (100%), while weak inhibition was seen in THP-1 cells (Figure 1). Based on the results of MTT assay, the concentration of AGT used in subsequent experiments was set at 50 μM. This was because no clear difference in results was seen between 50 and 100 μM. This concentration of AGT is comparable to 10 μg/mL (35 μM) used in the previous study using U937 cells.^7^

### Annexin V- and dead cell-positive rates

**Figure 2:**
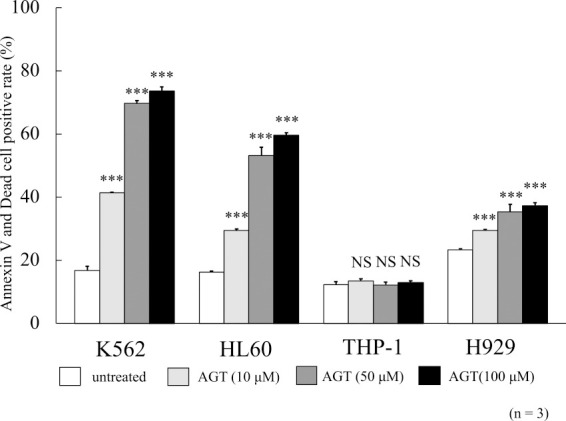
Rates of positivity for annexin V and dead cells after treatment for 24 h with AGT in four hematological tumor cell lines. These rates increased in K562, HL60, and H929 cells compared with those in untreated cells, but there was little increase in THP-1 cells. The results are expressed as mean±SD of three independent experiments (NS, *P<0.05, **P<0.01, ***P<0.001, compared with untreated cells).

The rates of positivity for annexin V and dead cells were evaluated after 24 h of incubation with AGT. These rates increased for K562, HL60, and H929 cells in the presence of AGT (Figure 2). However, the MTT assay results for THP-1 cells showed no increases in positivity for annexin V and dead cells. Since HL60 and K562 cells were rather more sensitive to AGT than H929 cells, the following studies were conducted with HL60 and K562 cells.

### Caspase-3/7 activity

**Figure 3:**
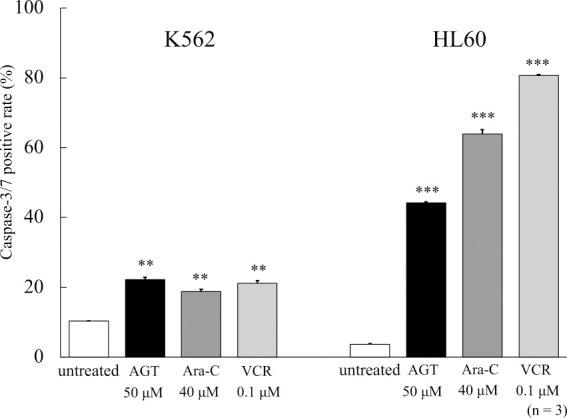
Caspase-3/7 activity after treatment for 24 h with AGT, Ara-C, or VCR in K562 and HL60 cells. Caspase-3/7 activity increased in K562 and HL60 cells compared with that in untreated cells. The results are expressed as mean±SD of three independent experiments (NS, *P<0.05, **P<0.01, ***P<0.001, compared with untreated cells).

In a previous study, we compared the antitumor effects of AGT with those of the anticancer agent Ara-C.^11^ In this study, we added VCR, which has a different antitumor mechanism than Ara-C, for comparison. Caspase-3/7 activity was evaluated after 24 h on K562 and HL60 cells treated with AGT (50 μM), Ara-C (40 μM), or VCR (0.1 μM). In both cells, caspase activity was increased after the addition of AGT compared with that in untreated cells (Figure 3); however, the increase of caspase-3/7 activity in K562 cells was much lower than in HL60 cells.

### Mitochondrial membrane depolarization

**Figure 4:**
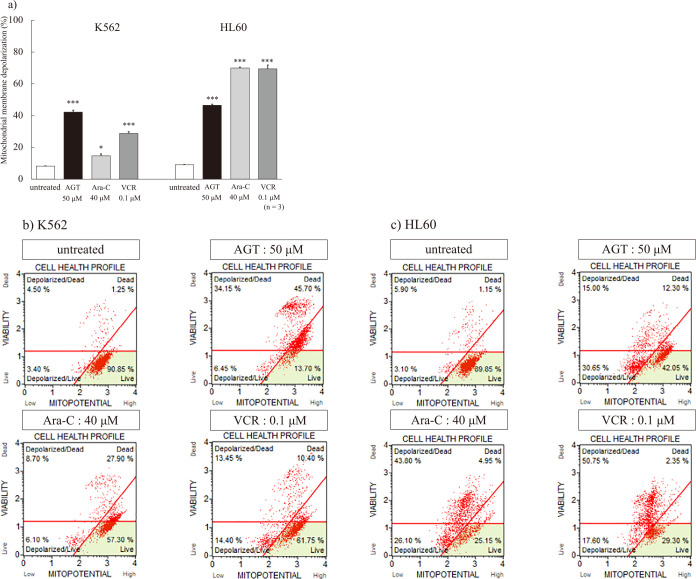
a　Mitochondrial membrane depolarization after treatment for 24 h with AGT, Ara-C, or VCR in K562 and HL60 cells. Mitochondrial membrane depolarization increased in K562 and HL60 cells compared with that in untreated cells. The results are expressed as mean±SD of three independent experiments (NS, *P<0.05, **P<0.01, ***P<0.001, compared with untreated cells). b, c　Dot plots of mitochondrial membrane depolarization.

After the addition of AGT (50 μM) or the anticancer agents, the mitochondrial membrane depolarization showed a clear increase compared with that of untreated cells (Figure 4a). HL60 cells were highly sensitive to the anticancer agents, but K562 cells showed higher sensitivity to AGT than to them. A dot plot of mitochondrial membrane depolarization is shown in Figure 4b, c.

### Cell cycle analysis

**Figure 5:**
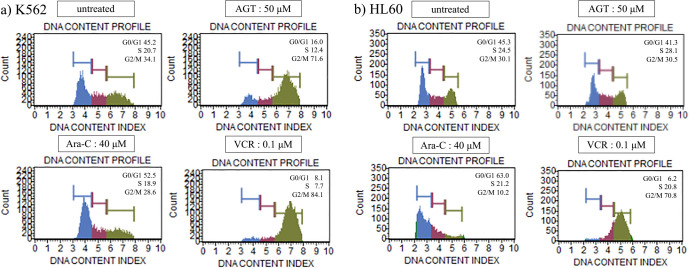
Cell cycle analysis after treatment for 24 h with AGT, Ara-C, or VCR in K562 and HL60 cells. The cell cycle was analyzed in terms of G_0_/G_1_ phase, S phase, and G_2_/M phase. The proportion of cells in G_2_/M phase was clearly increased compared with that of untreated cells for K562 cells, but there was no change for HL60 cells. The proportion of cells in G_1_ phase increased after treatment with Ara-C, while the proportion of cells in G_2_/M phase increased after treatment with VCR in K562 and HL60 cells.

After the addition of AGT (50 μM) to K562 cells, the proportion of cells in G_2_/M phase (71.6%) was clearly increased compared with that in untreated cells (34.1%), while there was no significant change in HL60 cells. In both cells, Ara-C and VCR caused increases in the proportions of cells in G_1_ and G_2_/M phases, respectively (Figure 5a, b).

### DNA fragmentation

**Figure 6:**
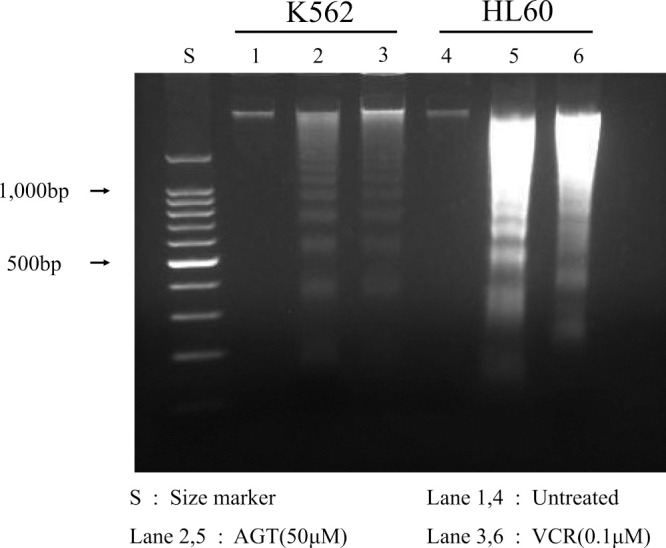
DNA fragmentation after treatment for 24 h with AGT or VCR in K562 and HL60 cells. DNA fragmentation was detected by agarose gel electrophoresis after treatment for 24 h with AGT or VCR in K562 and HL60 cells. Lane S, DNA size markers; lanes 1 & 4, untreated cells; lanes 2 & 5, treated with AGT; lanes 3 & 6, treated with VCR.

DNA fragmentation was detected by agarose gel electrophoresis on K562 and HL60 cells after 24 h of treatment with AGT (50 μM) or VCR (0.1 μM) (Figure 6).

### Expression of Bax and cytochrome c

**Figure 7:**
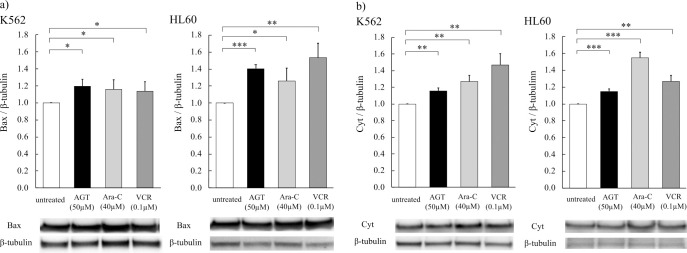
The expression of Bax and cytochrome c after treatment for 24 h with AGT in K562 and HL60 cells. The expression of Bax and cytochrome c after treatment for 24 h with AGT was increased in K562 and HL60 cells, as well as treatment with Ara-C or VCR, as determined by western blotting. The results are expressed as mean±SD of three independent experiments (NS, *P<0.05, **P<0.01, ***P<0.001, compared with untreated cells).

Increases of Bax and cytochrome c expression were observed after 24 h of incubation of K562 and HL60 cells with AGT (50 μM), Ara-C (40 μM), or VCR (0.1 μM), compared with the levels in untreated cells (Figure 7a, b).

## Discussion

Conventional cancer treatments include chemotherapy, radiation, and hormonal immunotherapy, but these treatments have serious side effects.^13^ Polysaccharides extracted from medicinal mushrooms, especially β-glucans, have been studied in vivo, in vitro, and in humans, and many clinical studies have been conducted to use these substances in combination with chemotherapy as a complement.^6^ The results have shown improved disease-free survival rates and health benefits for patients. Medicinal mushrooms have long been approved as an adjunct to standard cancer treatment in Japan and China, and have been reported to have an extensive clinical track record of safe use.^2^

We found that AGT from a diffusible fraction of hot water extract of ABM showed antitumor activity against leukemia cell lines U937, MOLT4, HL60, and K562,^7^ and revealed that AGT induces apoptosis in U937 cells.^11^ In this study, we examined the antitumor effects of AGT on K562, HL60, THP-1, and H929 cells to understand the mechanism behind AGT’s effects.

After the addition of AGT to four hematological tumor cell lines, there were clear effects of inhibiting cell viability as determined by the MTT assay and increases in the rates of positivity for annexin V and dead cells in K562, HL60, and H929, as in the case of U937 cells,^11^ while AGT had little effect on THP-1 cells. The reason for this difference is unclear, but THP-1 showed cell aggregation, indicating that AGT might affect the plasma membrane of the cells. THP-1 and H929 cells were reported to be sensitive to andrographolide (Andro), a terpenoid lactone isolated from the plant *Andrographis paniculata*, and Andro induced ROS-dependent apoptosis in THP-1 and H929 cells.^12^ Since Andro and AGT are hydrophobic and hydrophilic, respectively, the membrane acceptability of THP-1 to hydrophilic materials might differ from those of K562 and HL60. It would be important to identify the membrane receptor for AGT in K562 and HL60 cells.

In K562 and HL60 cells, we have shown that AGT induces caspase-3/7 activation, mitochondrial membrane depolarization, increased expression of Bax and cytochrome c, and DNA fragmentation. Bax is a proapoptotic member of the BCL-2 family and has been reported to interact with mitochondrial voltage-dependent anion channels (VDAC) to increase their opening, causing loss of membrane potential and release of cytochrome c. Cytochrome c and other apoptosis-promoting factors are released from mitochondria, causing the activation of caspases and finally inducing DNA fragmentation, a hallmark of apoptosis.^14^ Our results clearly indicate that AGT induces apoptosis in K562 and HL60 cells. To the best of our knowledge, no other reports of apoptosis induction by AGT have been published. This is the first report describing that AGT induces apoptosis by increasing mitochondrial membrane depolarization.

In cell cycle analysis after the addition of AGT, different results were obtained between K562 and HL60. An increase in the proportion of cells in G_2_/M phase was observed after the addition of AGT to K562, as in the case of VCR. However, no significant change in the cell cycle was observed in HL60 by AGT. Furthermore, AGT showed no effect on the cell cycle of THP-1 and H929 (data not shown). The action of AGT on the mitotic apparatus in K562 cells needs to be studied in detail.

Some reports of antitumor effects of extracts from ABM have been published. Blazein is a steroid [(22*E*)-6β-methoxy-5α-ergosta-7,22-diene-3β,5-diol] isolated from ABM, the effects of which on DNA fragmentation in various human cancer cells were studied. DNA fragmentation was observed in human lung LU99 and stomach KATOIII cancer cells after the addition of blazein.^15^ In addition, andosan, a commercially available mushroom extract containing 82.4% *Agaricus blazei* Murill extracted from mushroom mycelium, has been found to be cytotoxic to primary myeloma cells and other cells. Although the structure of andosan as well as its mechanism of action is unknown, it has been reported that, when andosan was added to myeloma cell lines, an increase in the proportion of cells in sub-G_1_ phase (DNA fragmentation) and a decrease in G_1_ phase were observed in cell cycle analysis.^16^ Hot water extract of ABM was also reported to be useful for the treatment of pancreatic cancer.^17^ The structural basis of this extract is unclear, but it significantly inhibited the growth of cultured pancreatic cancer cells through the induction of G_0_/G_1_ cell cycle arrest and caspase-dependent apoptosis against three human pancreatic cancer cell lines: MIAPaCa-2, PCI-35, and PK-8.

FA-2-b-β, an acidic RNA–protein complex extracted from wild edible ABM, induced apoptosis in CML K562 cells. The molecular target drug for CML is Glivec, a TK inhibitor. However, the main drawback of therapeutic TK inhibitors is the severe side effects seen in CML patients. Therefore, it is essential to investigate alternative drugs to minimize the side effects. In this context, it was reported that naturally occurring compounds could be used as new therapeutic targets in the management of CML.^18^

In this study, we examined the effect of AGT on four leukemia cell lines and found that it exerted strong antitumor effects, especially on K562, a chronic myelogenous leukemia cell line, and HL60, an acute promyelocytic leukemia cell line.

Imatinib, a tyrosine kinase inhibitor, has been used as molecular therapy for CML, showing significant efficacy. Imatinib is an ATP-binding antagonist that interacts with the P-loop/ATP binding site of the BCR-ABL protein; therefore, the presence of T315I mutation on the ATP binding site inhibits its function. Despite the high success rate of CML treatment with imatinib, it has been reported that a significant proportion of CML patients develop drug resistance due to mutations such as T315I.^19^ Against this background, AGT may be effective against CML drug resistance because it exerts an antitumor effect through a different mechanism of action than imatinib.

Acute promyelocytic leukemia (APL) is a type of acute myeloid leukemia (AML) associated with a strong tendency for bleeding. All-trans-retinoic acid (ATRA) was shown to induce functional and morphological maturation of APL cells, and ATRA monotherapy was used for treating APL with a high response rate, but the duration of response was short. Subsequently, the development of ATRA and chemotherapy combinations made APL a highly curable disease. Furthermore, arsenic trioxide (ATO, As_2_O_3_) monotherapy has been studied in patients with relapsed or refractory APL, for which complete remission was reported in over 80% of patients. However, it has been reported that 5%–10% of APL patients will relapse within the first 3 years.^20^ Since AGT has been shown to induce a high rate of apoptosis in HL60 cells, a promyelocytic leukemia cell line, it may have the potential to achieve clinical efficacy on APL patients.

Our present results suggest the possibility that AGT can function as an anticancer agent for certain types of hematological cancer cells. Further molecular-based analysis of AGT is necessary to elucidate its pharmacological effect of AGT.

## Conclusion

AGT showed antiproliferative effects on the hematological tumor cell lines K562, HL60, and H929, but showed no significant effect on THP-1 cells. In K562 and HL60 cells, AGT was shown to induce apoptosis via mitochondrial membrane depolarization.
